# Supplementary material for: Are carbon emissions trading and green financial instruments synergistic? -Comprehensive quantitative research based on content analysis
Source: PLoS One. 2024 Mar 7;19(3):e0298601. doi: 10.1371/journal.pone.0298601 (PMC10919601; doi:10.1371/journal.pone.0298601)
Supplement: S1 Data — (ZIP) [file pone.0298601.s001.zip › Data/地级市Z_F工作报告.zip!/╣π╢1⁄2/║╙╘┤╩╨/└┤╫╘╣1⁄2╓┌║┼í╛┬φ┐╦╩2╛▌═°í┐_2015.docx]

各位代表：

现在，我代表市人民政府向大会作政府工作报告，请予审议，并请市政协各位委员和其他列席人员提出意见。

2014年工作回顾

2014年，我市经济社会发展稳中有进、稳中向好。面对国内外复杂严峻的经济形势，特别是经济下行的压力，市政府在省委、省政府和市委的正确领导下，在市人大及其常委会和市政协的监督支持下，全面落实党的十八届三中、四中全会和习近平总书记系列重要讲话精神，积极有效应对困难挑战，全力以赴推动加快发展，坚定不移推进转型升级，较好完成了市六届人大四次会议确定的目标任务，经济社会发展取得了实实在在的成果。

一、经济发展实实在在

坚持经济建设第一要务，因势施策，干在实处，经济发展稳中有进。 主要经济指标争先进位。全市生产总值758.95亿元，比上年增长10.9%，增速在全省排第1；人均生产总值24881元，增长10%。规模以上工业增加值389.48亿元,增长18%。地方公共财政预算收入60.45亿元，增长23.9%，增速在全省排第1。固定资产投资453.29亿元，增长33.5%。实际利用外商直接投资2.26亿美元，增长7.1%；进出口总额39.5亿美元，增长22.4%。社会消费品零售总额265.01亿元，增长11.8%。城镇居民人均可支配收入18223元，增长10.9%。完成节能降耗和污染减排任务。 园区扩能增效成绩显著。“一区六园”投入开发建设资金39.44亿元，扩园12.6平方公里，完成工业增加值295.76亿元、增长30.4%，实现入库税收21.62亿元、增长18.1%。其中，“一大三小”转移园获评省优，占全省三分之一；争取资金6.63亿元，占全省四分之一。 现代服务业呈现新气象。生态旅游有新突破，万绿生态旅游产业园建设扎实推进，巴伐利亚庄园正式对外试营业，叶园温泉旅游景区获批国家4A景区，康泉十八、东江源温泉、佗城温泉度假区等一批重点旅游项目建设顺利，第十一届客家文化旅游节取得圆满成功，接待入市游客2201.47万人次、增长15.4%，旅游总收入177.01亿元、增长15.9%，旅游业由观光型向休闲型转变。商贸及电子商务有新气象，我市首个电商综合体百城百汇项目落户江东新区，市商业中心、汇景希尔顿逸林酒店等一批商贸项目成功开业。现代物流业有新进展，制定物流业发展规划，新出入境车检场启用，市高新区综合物流园和龙川铁路编组站物流园建设稳步推进。金融服务有新提升，各项贷款余额为699.01亿元，比年初增长22%，增速在全省排第2；金融机构向中小微企业贷款余额341.8亿元，占各项贷款余额的48.9%，有力促进了中小微企业发展。 科技创新能力增强。创建了河源广工大协同创新研究院，培育了一批科技创新平台，6家企业被认定为国家级高新技术企业。

二、项目建设实实在在

坚持项目第一抓手，以项目拉动投资，以项目带动发展。 “四个一批”、“三个50”和“三个100”工程成效凸显。新签约项目173个，同比增加84个；新开工项目162个，同比增加61个；新投产项目135个，同比增加40个，其中签约、开工、投产超3亿元项目达108个。新上规企业95家，新上限企业57家，新培育税收超千万企业72家，减产企业面由30.2%下降到18.1%，“三个100”工程两年目标超额完成。 “双百亿”工程强力启动。把实施“双百亿”工程作为转型升级的重要抓手，在谈、签约及在建10亿元以上的项目8个，其中开工项目4个，意向投资总额52亿元，为河源振兴发展注入了动力。 重点项目建设进展顺利。110个省、市重点项目完成投资180亿元，占年度计划110%。其中，省重点项目累计完成投资89.5亿元，占年度计划118.4%。

三、基础设施建设实实在在

千方百计攻坚克难，全力推进以交通为重点的基础设施建设。 交通设施不断完善。完成交通基础设施投资66.8亿元，增长40%。高速公路建设全面推进，汕湛、大广高速公路河源段加快推进，汕昆高速公路河源段开工建设。完成粤赣高速公路城北和东源出入口连接线升级改造，推进埔前出入口互通立交改建工程。完成国省道改造里程60公里，县乡公路改造里程100公里，新农村公路路面硬化595.5公里。加快建设大环市路。城南客运站动工建设。武深、河惠莞高速公路河源段前期工作进展顺利，赣深客运专线河源段完成预工可报告编制，龙汕铁路、杭广铁路启动预工可工作。启动河源通用机场规划选址工作。 能源设施建设加快。国家西气东输三线、省天然气管网三期、全市天然气主管网、河源电厂二期等工程开展前期工作。加快电网设施建设，建成投产110千伏及以上主网项目7项，新增主变容量19万千伏安，解决重过载配变电站244个、低电压台区128个，供电能力得到提高。启动市区天然气置换工作。市区水源工程稳步推进。

四、提质扩容实实在在

着力推进中心城区提质扩容，完善城市功能，增强辐射带动能力，城市宜居水平进一步提高。 新区建设全面启动。组建了江东新区管理机构，完成了新区城市总体规划和专项规划，编制了控制性详细规划。大胆创新社会化的管理服务模式，积极探索多元化的融资经营模式。争取各类资金支持15亿元，完成土地征收2平方公里。在谈、签约项目40多个，意向投资达300亿元。 市政功能不断完善。市区迎客大桥建设加快，滨江大道二期基本建成。完成新城中西片区13条市政道路和沿江路等升级改造。完成老城太平古街等18处易涝路段整改，解决重点区域“水浸街”问题。贯通东江西路等一批“断头路”，缓解市民出行难问题。72项老城区背街小巷改造工程顺利推进，完善老城区市政设施。启动国家园林城市创建工作，建成客家文化公园二期、七寨湖公园、红星公园，改造提升九重门等一批公园，完成市区道路绿化升级改造。完成市区两江四岸和全民健身广场亮化工程，城市变得更亮更美。 城市管理得到加强。实现市区建设用地控规全覆盖。建立健全土地长效管理机制，通过国家土地例行督察，用地保障更加有力。大力整治城市“六乱”，提升城市管理综合执法水平和保洁水平，国家卫生城市成果得到巩固。推进公共交通建设，一批新公交站点、新能源公交车投入使用。

五、“三农”工作实实在在

充分激发农业农村发展活力，切实提升农民生活水平。完成农林牧渔增加值89.98亿元，实现农民人均可支配收入9867元，分别增长4.3%和12.3%。 灯塔盆地建设亮点突出。完成核心区规划编制。争取中央和省扶持资金1.1亿元。荣获全国2013年农业改革与建设试点绩效评价第3名。与5所科研院所、大专院校签订合作协议，引进8支科研创新团队，被认定为国家农业科技创新与集成示范基地。 农业现代化进程加快。以农村综合改革和农用地流转改革为契机，推动农业规模化、现代化发展。新增农民专业合作社888家，辐射带动农户26万户，户均增收3100元以上。高标准基本农田建设加快，耕地保护工作得到加强。农村土地经营权确权颁证试点工作稳妥推进。河源活猪基地被认定为省级外贸转型升级专业型示范基地。 新农村建设展现新貌。启动2个名镇、26个名村、66个示范村建设，7个镇入选全国重点镇，龙川县启动中央苏区县幸福村居示范县创建活动。村级公益事业“一事一议”成效明显，获得省级以上财政资金4726万元，建设913个项目，进一步改善了农村基础设施。移民区生产生活条件持续改善。 农田水利建设有序推进。全面实施最严格水资源管理制度。完成东源、紫金2个水利示范县和东源、紫金、和平3个小农水重点县项目年度建设任务，较好完成中小河流治理、病险水库除险加固、小流域综合治理和机电排灌等工程建设。源城区实现村村通自来水。 扶贫开发成效显著。新一轮扶贫开发扎实推进，投入资金14.79亿元，平均每村522.6万元；启动扶持项目6082个，平均每村21.49个；改造低收入住房困难户住房2.46万户，超额完成省下达的任务；“两不具备”村庄搬迁安置1691户；2.44万户贫困户、11.23万贫困人口实现脱贫，超额完成省下达的减贫任务。

六、对口帮扶实实在在

抢抓机遇、主动对接，积极推进对口帮扶工作。 共建园区有序推进。深河产业城共建顺利，深圳特区建设发展集团参与开发建设，编制了总体规划，规划总面积11.6平方公里，计划总投资130亿元。深圳对口帮扶资金到位5.5亿元。启动综合保税区和无水港规划。 招商引资成效明显。两市合办3场招商推介会，引进项目101个，总投资688亿元；其中签约项目69个，投资435亿元；动工项目19个，投资140亿元。同时，全方位开展了交通、教育、卫生、文化、人才等领域的对口帮扶工作。

七、民生事业实实在在

顺应群众所思所盼，切实提升群众的幸福感。 教育创强步伐加快。全市新增18个教育强镇，东源县成功创建省教育强县，源城区实现教育强镇全覆盖。创建义务标准化学校85所，覆盖率提升到89.1%。市区公园西小学、碧桂园学校建成，市一中高中部、高新区实验学校、市直机关幼儿园、高莲小学等顺利推进。全面实施“振兴初中”计划，落实山区和农村边远地区义务教育学校教师岗位津贴，致力提高教育教学水平。成功创建河源技师学院和3所国家中职示范学校。 社会保障水平提升。新增就业岗位、安置失业人员和就业困难人员再就业以及创业带动就业人数均超额完成任务，城镇登记失业率控制在2.44%以内。完成城乡居民医疗保险参保工作，城乡居民社会养老保险和城乡基本医疗保险参保率达99%，完成低保和五保提标工作。保障性住房建设任务和因灾全倒户重建工作完成。物价水平保持稳定。残疾人事业稳步发展。 卫计工作持续发展。全面推行县级公立医院改革，巩固完善基本药物制度和基层医疗卫生机构运行新机制。落实重大疾病防控工作。实行城乡居民大病保险、医保普通门诊统筹、医保门诊特定病种三项制度。市人民医院二期、市妇幼保健院二期、市精神卫生防治中心建设稳步推进。实施“单独二孩”政策，完成人口计划，稳定低生育水平。 “文化河源”加快推进。成功获得全国文明城市提名资格。市图书馆新馆、博物馆、档案馆建设加快。新建、改扩建乡镇（街道）综合文化站40间，建成乡镇（街道）综合文化站阅览室19间、村（社区）电子阅览室125间。各县区数字影院建成投入运营。第一次全国可移动文物普查工作顺利推进。完成乡镇全民健身工程建设任务，市体校迁建顺利开展，参加省第五届少数民族传统体育运动会取得历史最好成绩。 社会保持和谐稳定。扎实推进社会治安综合治理和平安建设工作，开展公安“六大专项”行动，各类刑事案件破案数上升28%，八类严重暴力案件立案数下降27.3%、破案率上升10.7%，群众安全感居全省第5名。积极开展普法和人民调解，完成一村（社区）一法律顾问试点，信访维稳工作得到加强。扎实开展省社会体制改革试点。基层社会治理创新“四个一”工程深入推进，社会组织加快培育发展。落实安全生产“一岗双责”制度，开展“打非治违”专项行动，安全生产形势总体稳定。产品质量和食品药品安全监管、应急管理等工作得到加强。深入开展严打“三偷”行动。国防和武装工作创新发展，遂行急难险重任务能力提高。 “十件实事”全面完成。投入资金35.6亿元，“十件实事”实现预期目标，群众“看病难”、“上学难”、“出行难”等问题得到缓解。

八、生态保护实实在在

致力改善提升生态优势，主要生态保护指标名列全省前茅。 加大投入治水。全力推进47个新丰江水库生态环保项目建设。污水处理厂建成5个、在建6个。完成东埔河白岭头黄子洞段整治工程。大力开展东江水环境综合治理，东江水质保持稳定。建立河源与赣州水污染联防联控机制。 抓住重点控污。加大大气污染整治力度，积极实施“五大治污工程”，空气主要污染物达标。中心城区雨污分流规划建设加快。农业面源污染治理工作加强。 全民动手添绿。完成森林碳汇重点生态工程建设任务91.24万亩，建设生态景观林带69公里，生态景观林带补植套种178.35公里，建设乡村绿化美化示范村69个。加大森林资源管护力度，推动治采复绿，复绿面积1127.5亩。新增森林公园25个、湿地公园3个。

九、政府建设实实在在

大力加强政府自身建设，以实际行动取信于民。 作风建设成效凸显。以党的群众路线教育实践活动为契机，进一步提升行政效能。严格落实中央八项规定，坚决遏制“四风”，创新开展政风行风评议活动，党员干部自律意识明显增强，良好政治生态逐渐形成。市地税局荣获“全国五一劳动奖状”。 深化改革扎实推进。新一轮政府机构改革基本完成，行政审批事项再压减10%，平均每件事项缩短审批时限13天。在全省率先全面铺开标准化建设工作，市直39个单位共440项行政审批事项编写了办事指南。财税制度改革有序推进，全市财政预决算和“三公”经费信息全面公开，开展了全口径政府预算编制工作，地方政府债务管理防控机制不断完善，“营改增”改革试点扩面。商事制度改革掀起社会创业热潮，全市登记各类市场主体突破10万户。稳步推进国企改革。 政务效能得到提高。认真开展“三赛”活动，建立“马上办”工作机制，及时破解项目“卡壳”问题。网上办事大厅建设提前一年完成，实现了省、市、县、镇、村五级互联互通，网上办结率51%，高出省要求10个百分点。人大代表建议、政协提案办复满意和基本满意率100%。完成了村委会、居委会换届选举工作。

此外，妇女儿童、法律援助、民族宗教、人民武装、“双拥”创建、优抚安置、防灾减灾、驻外机构、外事侨务、机关事务、殡葬管理、地方志、粮食、人事、统计、审计、对台、老龄、人防、气象、供销、打私等各项工作取得新进展。

各位代表！上述成绩来之不易，值得珍惜。这是省委、省政府和市委正确领导的结果，是全市人民共同奋斗的结果，也离不开深圳市的全面对口帮扶和广州、中山等兄弟市以及海内外各界人士的关心支持。在此，我代表市人民政府，向全市广大干部群众、驻河源部队、武警、消防官兵和各民主党派、人民团体、社会各界人士致以崇高的敬意！向关心支持我市改革发展的兄弟市以及港澳同胞、台湾同胞、海外侨胞表示衷心的感谢！

看到成绩的同时，我们也清醒地认识到，我市经济社会发展还存在不少困难和问题。主要表现在：受宏观经济下行等大环境影响，生产总值、规上工业增加值等经济指标未能实现预期目标；资源紧缺与低效利用并存，制约项目落地的短板没有得到根本性改变；好项目、大项目的数量不多、体量不大；传统竞争优势在不断弱化，招商引资资源和政策亟待整合；产城融合不够，产业化与城市化未能实现良性互动发展；基础设施建设与城市管理相对滞后，交通拥堵、城市“六乱”等问题依然存在；医疗、社保、教育等民生工作，与老百姓的期盼仍有较大差距；一些公职人员的服务意识和履职效率有待提高，依法行政水平需要加强。对此，我们将高度重视，采取措施，认真予以解决。

2015年工作安排

2015年是全面完成“十二五”规划的收官之年，是全面深化改革的关键之年，是全面推进依法治国的开局之年，做好今年工作意义重大。总体来说，我市仍处于大有可为的发展上升期，实现振兴发展有不少积极因素，但宏观经济下行压力依然较大，必须付出艰辛努力。我们要审时度势、因势施策，牢牢把握发展主动权，确保经济社会平稳健康持续发展。

今年政府工作的指导思想是：全面贯彻党的十八大、十八届三中、四中全会、中央经济工作会议、省委十一届三次、四次全会精神，全面落实市委六届七次全会决策部署，主动适应经济发展新常态，紧扣“三个定位、两个率先”和振兴发展的总目标总要求，突出“三大抓手”，坚持“稳”字当头，保持经济平稳健康发展，做到调速不减势、量增质更优；坚持“实”字为主，一切从河源的实际出发，以求真务实的精神和真抓实干的作风推动既定战略的实施；坚持“好”字优先，推动经济社会提质增效，努力完成“十二五”规划，为河源振兴发展奠定基础。

今年全市经济社会发展的主要预期目标是：生产总值增长11.5%；规模以上工业增加值增长17%；地方公共财政预算收入增长17%；固定资产投资增长20%；社会消费品零售总额增长12%；外贸进出口总额增长5%；居民消费价格指数涨幅控制在3%以内；全体居民人均可支配收入增长11%；人口自然增长率控制在8‰以下；完成节能减排任务。

为实现上述目标，重点做好九大方面工作：

一、在壮大实体经济上下功夫，筑牢河源振兴发展根基

做强实体经济，提升经济质量，巩固和保持经济加快发展的良好势头。

突出主导产业做大工业总量。继续实施“四个一批”、“三个50”、“三个100”工程，两年培育新上规、新上限企业各100家；以十亿企业、百亿产业为目标，大力实施“双百亿”工程，着力培育食品饮料产业、新材料产业成为百亿级产业集群。重点培育扶持新一代移动通讯终端设备、太阳能与光伏应用、钨矿精深加工、食品饮料等特色优势产业。

打造特色园区带动产业集聚。鼓励社会资本以BT、BOT、PPP等方式参与园区建设，逐步建立政府主导、多方参与、市场运作的开发模式。加强园区公共配套和产业配套建设，提升产城融合水平。加大招商引资工作力度，坚持全市招商一盘棋，大力整合资源和政策，实施园区产业链招商，创新招商模式，提升引资实效。推动市高新区创建国家级高新区。力争全市工业园区扩容10平方公里以上，园区规上工业增加值增长22%以上，固定资产投资增长26%以上。

做大民营经济扶持骨干企业。完善扶持民营经济发展政策，健全中小微企业公共服务体系，扶持民营企业发展，充分发挥民营经济的富民效应。大力扶持重点企业、龙头企业，在资源配置、优惠政策上给予倾斜,鼓励增资扩产、技改创新、做大品牌、上市发展，助推企业向10亿元、50亿元、100亿元规模迈进，切实发挥龙头企业的带动作用。

抓住重点项目增强发展后劲。充分发挥投资拉动作用，扎实推进总投资1326亿元、年度投资242亿元的98个省市重点项目建设。结合“十三五”规划编制，谋划储备一批项目，夯实经济发展基础。

打造生态休闲旅游目的地。加快万绿生态旅游产业园建设，做好第二期扶持资金的申报工作。继续推进市旅游景观大道、太平古街等一批景观道路建设，完善旅游交通指示牌，加快智慧旅游建设。推进重点旅游项目建设，支持启动巴伐利亚庄园二期建设，加快康泉十八、东江源温泉等重点旅游项目建设。扶持建设一批特色景观旅游村镇，引导和规范农家乐发展。办好第十二届客家文化旅游节，精心包装和策划旅游精品线路，打响生态休闲旅游品牌，使河源成为有吸引力的旅游目的地。

积极推进对口帮扶工作。加快深河产业城“双港双谷一区”开发建设，完成“三通一平”2平方公里以上，实现动工项目合同投资100亿元以上。推进中兴通讯（河源）基地建设。引导和支持深圳金融机构、大型企业、民间资本参与我市经济社会建设。
